# Spine Centers in Northeastern United States: Assessing Similarities and Disparities to Build more Homogeneity Across the Country

**DOI:** 10.1007/s11916-026-01538-9

**Published:** 2026-08-10

**Authors:** Zachary Vazquez, Sabrina L. Zeller, Ankita Jain, Eris Spirollari, Ariel Sacknovitz, Matthew Merckling, Ethan Parisier, Sima Vazquez, Donald MacElroy, John K. Houten, John V. Wainwright, Sayed E. Wahezi, Alaa Abd-Elsayed, Ugur Yener, Hatice Begum Ciftci, Merritt D. Kinon

**Affiliations:** 1 School of Medicine, New York Medical College, Valhalla, NY 10595 USA; 2 Brain and Spine Institute, Westchester Medical Center at New York Medical College, 100 Woods Road, Macy Pavilion, Valhalla, NY 10595 USA; 3 Department of Neurosurgery, Westchester Medical Center at New York Medical College, Valhalla, NY 10595 USA; 4 Department of Neurosurgery, Mount Sinai Hospital, New York, NY 10029 USA; 5 Department of Neurosurgery, Miller School of Medicine, University of Miami, Miami, FL USA; 6 Department of Physical Medicine and Rehabilitation, Montefiore Medical Center, Bronx, NY USA; 7 Department of Anesthesiology, University of Wisconsin, Madison, WI USA

**Keywords:** Spine center, Centers of excellence, Spine care, Spine accreditation, Spine surgery, Spine certification, Spine center guidelines

## Abstract

**Propose of Review:**

Spine centers are common, but no standardized regulations govern their designation. Existing certifications are issued by multiple independent organizations and are not necessarily equivalent, while many centers remain non-accredited. Consequently, centers may vary in qualifications, quality of care, and services. This study examined and compared the defining characteristics of spine centers in Northeast America.

**Recent Findings:**

A total of 194 spine centers were identified and compared based on accreditation, services, provider numbers, and proximity to metropolitan areas. Accreditation requirements from The Joint Commission (TJC), Det Norske Veritas (DNV), Aetna, and Blue Cross Blue Shield (BCBS) were analyzed. While substantial overlap existed, Aetna and BCBS largely built upon TJC standards, whereas DNV requirements differed, and exact criteria varied across organizations. Overall, 56% of centers were accredited. Compared with non-accredited centers, accredited centers more frequently employed neurosurgeons, offered multidisciplinary care, and were affiliated with academic medical centers. Logistic regression demonstrated that employment of neurosurgeons and academic affiliation independently predicted accreditation. Accredited centers also tended to be closer to metropolitan areas and employed more providers. However, rates of non-surgical treatment, minimally invasive surgery, and physical therapy services were similar regardless of accreditation status.

**Summary:**

Although accredited centers reported certain characteristics at higher rates, the variability between accreditation requirements and similarity between accredited and non-accredited centers could confuse prospective patients. While this study provides a preliminary profile of Northeast spine centers, our findings reinforce the need for standardized guidelines for spine center status.

**Supplementary Information:**

The online version contains supplementary material available at 10.1007/s11916-026-01538-9.

## Introduction

Managing excessive healthcare costs is an increasingly urgent problem in the United States (US), where Americans spent $4.5 trillion on healthcare in 2022 alone [1]. These large expenditures are not, however, made efficiently, with some estimates placing nearly 25% of all healthcare spending in the form of low-value care, poor care coordination, and administrative bloat [2]. One strategy in combating inefficiencies is the development of centers of excellence (COE). These interdisciplinary programs seek to identify and encourage centers that maximize provision of high-value care, control costs, and improve patient outcomes, and spinal care has been the focus of many of these efforts, with some early research indicating that they may reduce costs while preserving or improving the quality of care [3, 4].

However, facilities that call themselves “spine centers” are not subjected to the same regulatory standards as other forms of COE [3–6]. Several independent organizations certify spine centers, but there is no specific accreditation required for a facility to identify itself as a “spine center” [7]. This has led to a lack of standards for what constitutes a spine center. Existing spine centers can vary in size, characteristics, and scope of practice, ranging from large multidisciplinary care centers affiliated with academic institutions to single doctors working with external practitioners. The resulting heterogeneity of practice sites presented to the public as “spine centers” is thus confusing to patients seeking reliable spinal care, insurance providers seeking to standardize payments for spine center services, and practitioners seeking employment in spine-related care.

This study aims to analyze the characteristics of spine centers in the Northeastern United States, stratified by formal accreditation status, to identify key similarities among existing centers and to develop a profile to identify and designate future centers.

## Materials and Methods

### Data Collection

We identified existing spine centers in the Northeastern US (Connecticut, Delaware, Maine, Maryland, Massachusetts, New Hampshire, New Jersey, New York, Pennsylvania, Vermont, and Rhode Island) using the American Hospital Directory, Blue Cross Blue Shield’s (BCBS) Blue Distinction Center (BDC) Facility Finder, Aetna’s Ortho Institute of Quality (IOQ) facility listing, Quality Check by The Joint Commission (TJC), Det Norske Veritas’ (DNV) reports of awards of Advanced Certification in Orthopedics and Spine, and internet listings for spine centers on Google Maps [8–11]. Facilities representing themselves as spine care centers, spine and pain centers; spine institutes, and other variations of the phrase “spine center” were included. Facilities were also included if they had departments affiliated with spine care, spine surgery, brain and spine care, spinal neurosurgery, or any spine program, or an orthopedics/neurosurgery department specializing in spinal treatment. Facilities that did not provide spine surgery were excluded.

Key characteristics of spine centers included achieving accreditation, affiliation with research and/or academic medical centers, services provided, number of providers (including surgeons, neurologists, physiatrists, and other physician specialties), specialties of affiliated providers, number of campuses, and location of the main campus. The websites of identified spine centers were surveyed to obtain information about each of the above characteristics.

Non-surgical services included any treatments other than surgery, such as physical therapy, rehabilitation medicine, epidural injections, and chiropractic care. Neurological surgeon participation signified the inclusion of at least one neurosurgeon among a center’s providers. Multidisciplinary care indicated collaboration between orthopedic and neurological surgeons (though spine centers also employed neurologists, physiatrists, physical therapists, and others) and was indicative of a center’s stated holistic approach to treating spinal conditions.

The overland distance from each center to the center of the nearest major population center (MPC), defined as a city within a major metropolitan area as designated by the US Census’ Principal Cities of Metropolitan and Micropolitan Statistical Areas, July 2023, was determined using Google Maps’ in-application measurement tool. Centers of MPCs were used as endpoints rather than perimeters to minimize potential over- or underestimates of distance resulting from irregular MPC borders, along which arbitrary endpoints would need to be defined for each spine center. Similarly, absolute overland distance was obtained rather than driving distance to maximize replicability.

### Statistical Analysis

Data was collected in Excel and analyzed using Stata, v. 18. Descriptive statistics, including means and standard deviations (SD) or medians and interquartile ranges (IQR) for continuous variables, and frequencies and proportions for categorical variables, were reported. Differences between accredited and non-accredited centers were assessed using the two-sample t-test for normally distributed continuous variables and the Wilcoxon Rank Sum Test for non-normally distributed continuous variables. Pearson’s Chi-square Test was used to identify differences between categorical variables, and odds ratios with 95% confidence intervals were reported for characteristics that differed at statistically significant levels. Fisher’s exact test was performed to compare nonparametric categorical data.

We used forward stepwise regression to build a multivariate model to extend our examination of the differences leading to a center being accredited or not. Self-reported accreditation status was used to stratify groups. A multivariate logistic regression model was fitted to examine the impact of several covariates of interest on accreditation status. A forward selection procedure was then applied to identify the most significant predictor variables for accreditation status. Statistical significance for all analyses was determined using a two-tailed alpha level of 0.05.

The following organizational accreditation standards were also examined: Core and Advanced Certification in Spine Surgery (ACSS) from TJC; BDC and BDC+ (Blue Distinction Center+) from BCBS; Spinal IOQ from Aetna; and Orthopedic and Spine Center of Excellence Designation and Advanced Spine Surgery Certification (ASSC) from DNV.

## Results

### Accreditations

We reviewed 194 spine centers in the Northeastern US. Of the total, 108 (56%) centers reported accreditation from at least one of the four selected organizations. Of these, 29 (27%) were accredited by TJC, 25 (23%) by Aetna, 85 (79%) by BCBS, and 1 (1%) by DNV. The remaining 86 (44%) reported no formal accreditations.

### Spine Center Characteristics

Categorical characteristics reported by spine centers were normally distributed and are represented in Table 1. Ninety accredited centers (83%) had neurosurgeons, compared with only 51 non-accredited centers (59%; *p* < 0.001). Similarly, 64 (59%) accredited centers reported providing multidisciplinary care, whereas only 35 (41%) non-accredited centers did so (*p* = 0.010).

**Table 1:** Proportion of accredited and non-accredited spine centers reporting service characteristics

Characteristic *n* (%)	Total Centers 194 (100%)	Accredited Centers 108 (56%)	Non-Accredited Centers 86 (44%)	*P*-value
Non-surgical Treatments	133 (69%)	68 (64%)	65 (76%)	0.073
Minimally Invasive Surgical Options	137 (71%)	79 (73%)	58 (67%)	0.4
Robotic-Assisted Surgery	35 (18%)	20 (19%)	15 (17%)	0.8
Affiliated Neurological Surgeons	141 (73%)	90 (83%)	51 (59%)	**< 0.001**
Affiliated Orthopedic Surgeons	144 (74%)	80 (74%)	64 (74%)	> 0.9
Multidisciplinary Collaboration (Orthopedic and Neurological Surgeons)	99 (51%)	64 (59%)	35 (41%)	**0.010**
Affiliated Physical Therapists	114 (59%)	64 (59%)	50 (58%)	0.9
Research	92 (47%)	55 (51%)	37 (43%)	0.3
Academic Center	126 (65%)	80 (74%)	46 (53%)	**0.003**

The majority of both accredited and non-accredited centers offered non-surgical treatments (76%; *p* = 0.073), care from orthopedic surgeons (74%; *p* > 0.9), and physical therapy (59% and 58%, respectively; *p* = 0.9) at similar rates. The same can be said of institutional involvement in research (51% of accredited and 43% of non-accredited centers, *p* = 0.533), with approximately half of centers in each group hosting studies. Robotic-assisted surgery was offered by a minority of centers in both the accredited and non-accredited groups (19% in the accredited group and 17% in the non-accredited group; *p* = 0.8). The majority of both accredited and non-accredited centers (73% and 67%, respectively; *p* = 0.4) offered minimally invasive surgical procedures.

**Fig. 1:**
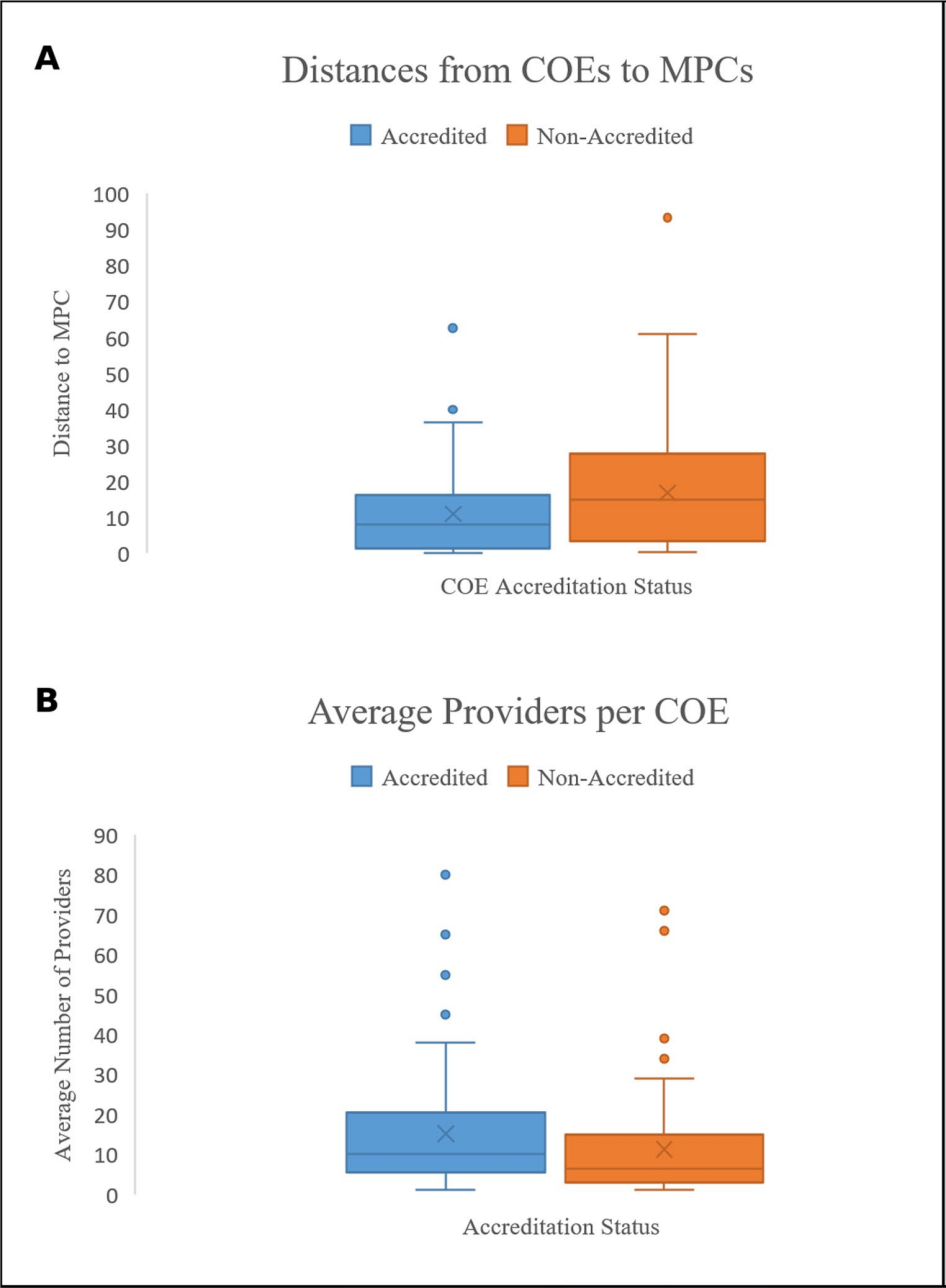
Distribution of spine-center characteristics: (**A**) distances from COEs to MPCs and (**B**) average number of providers per COE. A. Distribution of spine center distance to nearest MPC. Center distances calculated using Google Maps were compiled, upper and lower bounds identified, and interquartile ranges calculated. B. Distribution of average number of spine center providers. Provider numbers were compiled, upper and lower bounds identified, and interquartile ranges calculated

There was high intra-group variability in distance from MPCs across centers, and the data was non-parametrically distributed (Table 2). Over 50% of all centers fell within 20 miles of an MPC, and over 75% fell within 40 miles (Fig. 1A). Accredited centers were closer to MPCs by an average of 5.85 miles (*p* = 0.003). Data on the average number of providers was similarly non-parametrically distributed, with high intra-group variability (Fig. 1B). Accredited centers had an average of 2.59 additional providers than non-accredited centers (*p* = 0.027), but 75% of all centers had an average of 15 providers or more (Table 2).

**Table 2:** Continuous characteristics and multivariable predictors of accreditation. Panel A compares distance from major population centers and number of providers; panel B presents the multivariable logistic regression model. Statistically significant association (p < 0.05) is identified between accredited status and both distance from major population centers and number of providers employed. *OR* Odds ratio, *CI* confidence interval

Panel A			
Accreditation Status (*n*)	Accredited (108)	Non-Accredited (86)	*P*-value
Distance (miles)	11.03 ± 12.07	16.86 ± 15.82	**0.003**
Providers	14.80 ± 14.90	11.21 ± 12.91	**0.027**
Panel B			
Characteristic	**OR**	**95% CI**	**P-value**
Neurosurgery			**0.002**
No	-	-	
Yes	3.23	1.55, 6.92	
Teaching			**0.029**
No	-	-	
Yes	2.13	1.08, 4.25	
Miles to MPC	0.98	0.95, 1.00	0.055
Non-Surgical			0.060
No	-	-	
Yes	0.50	0.24, 1.03

Regression analysis (Table 2, Panel B) was performed to identify the characteristics most likely to predict a center’s formal accreditation. The strongest positive predictors of accreditation were employment of neurosurgeons (OR: 3.23 [1.55, 6.92]; *p* = 0.002) and affiliation with an academic medical center (OR: 2.13 [1.55, 6.92]; *p* = 0.029). While accredited centers were closer to MPCs on average, the distance to an MPC was not predictive of a center’s accreditation status (*p* = 0.055). Offering non-surgical care options was associated with a 50% reduction in the odds of achieving formal accreditation, though this finding was not statistically significant (OR: 0.5 [0.24, 1.03]; *p* = 0.06).

### Accreditation Requirements

Common accreditation requirements across all organizations included continuity of care, with an emphasis on collaboration between providers beginning in pre-surgical consultation and continuing through post-surgical rehabilitation; achievement of a specific patient/surgical volume; and satisfactory results of an on-site review. Here, we include a summary of prominent accreditation requirements by organization. Full requirement details for each organization can be found in their respective documentation.

The specific requirements (Supplementary Table 1) varied somewhat between organizations. Notable differences included BDC, ASSC, and IOQ requiring prior certification. In contrast, IOQ required a spinal surgery-related accreditation; BDC and ASSC did not specify that eligible accreditations were restricted to spine treatment. IOQ and BDC further differentiated themselves by requiring provider and facility affiliation with their insurance networks. Because of Aetna’s relatively few requirements beyond prior accreditation, the IOQ designation did not distinguish itself as an independent certification as much as BDC/BDC+, which expanded upon the requirements of other organizations’ accreditations with their own performance-related criteria. ASSC was unique for explicitly requiring a staff safety system to report “near misses, medical errors, and adverse events” [9]. It also had the most extensive list of original requirements for any singular center’s application, going into the greatest level of detail about both the operational and bureaucratic organization of a center’s spine program.

## Discussion

The definition of a “spine center” is not regulated at a national level, nor is the designation of “spine center” status officially made by any singular, central entity. This study aimed to identify the characteristics of spine centers in the Northeastern US, compare centers with and without existing spine care accreditations, and elucidate the features that make a facility a “spine center”.

Spine centers in the Northeastern US are defined by two primary methods: certification in spinal care from accrediting organizations and self-reported characteristics. Over the course of this study, partial overlap was found between these methods. Five of the characteristics most commonly reported by centers were significantly more common among accredited centers than among non-accredited centers. These included participation of neurological surgeons, collaboration between orthopedic and neurological surgeons, affiliation with an academic medical center, distance to the nearest MPC, and number of providers. However, our logistic regression model found that only neurosurgeon participation and academic center affiliation increased the odds that a center was formally accredited. This raises the question of why only these two characteristics are more strongly associated with accreditation.

There were numerous similarities in accreditation requirements across the reviewed organizations, primarily regarding patient outcomes after surgery and handoffs between members of the perioperative team. Key differences appeared to derive from the reported thoroughness of program review; DNV’s ASSC stood out for its more in-depth analysis of program characteristics, requiring more information than any other for certification procedures. However, TJC’s ACSS also distinguished itself by requiring quarterly rather than annual or tri-annual reporting of performance metrics. BCBS and Aetna both differed from TJC and DNV in that they required prior certification with one of the latter organizations to be eligible for BDC/BDC + and IOQ certification, respectively. Thus, it could be said that they were the most thorough in their program evaluation, implicitly incorporating the aforementioned organizations’ accreditation requirements into their own. They could also be considered more selective, given that they reserved certification only for programs affiliated with their respective provider networks. Yet, in the case of Aetna, this also raises concerns over the validity of the IOQ certification being distinct from ACSS or ASSC. Unlike BCBS, Aetna’s only additional requirement appears to be that program providers are affiliated with the Aetna insurance network.

Accreditation requirements, however, do not appear to mention either of the two identified predictive characteristics. In fact, no certification explicitly requires neurosurgical participation in a center. By comparison, DNV’s ASSC requires that “every patient is under the care of [an MD or DO] privileged in orthopedic surgery and/or spine surgery,” and “spine surgeons” are mentioned in both ASSC and ACSS guidelines. However, they do not specify which specialty (or specialties) qualify for this title. The emphasis that these organizations place upon orthopedics appears consistent with the marketing of their accreditations. TJC promotes ACSS as an “Orthopedic Certification,” while DNV claims that the ASSC “[is] designed to recognize excellence in an organization’s Orthopedic and/or Spine Surgery programs.” Since BCBS’s BDC and Aetna’s IOQ certifications both require either ACSS or ASSC certifications without further specifications about provider specialties, these accreditations naturally inherit the same emphasis on orthopedics. Thus, it appears that the orthopedic focus these organizations have assigned to their accreditations has shifted attention to orthopedic surgeons in centers, contrary to our findings about the positive impact of neurosurgeon participation on formal accreditation.

Conversely, it is surprising that multidisciplinary care provision would have no impact on accreditation likelihood as suggested by our logistic regression model. Given that ASSC, BDC, and ACSS require interdisciplinary collaboration, it would be easier for centers that feature interdepartmental collaboration between orthopedic and neurological surgeons to obtain these accreditations. However, in the current healthcare system, there has been a universal emphasis on multidisciplinary care, which may be why this feature is not predictive of formal accreditation [12–14].

Furthermore, other factors that enhance centers’ ability to meet accreditation requirements may also contribute to the reporting of neurological surgeon participation and academic center affiliation. For instance, centers affiliated with large, urban hospitals (closer to MPCs) may more easily meet accreditation requirements for patient or surgical volume and draw on physicians from multiple specialties within the hospital network. This may also explain why centers featuring neurosurgeons and affiliated with academic centers are more likely to be accredited: having access to a large network of providers would undoubtedly make it easier to involve neurological surgeons in a center, and the setting of a high-volume urban health network would likewise be ideal for a residency training program. Additionally, centers in such settings may be more likely to seek accreditation to distinguish themselves from neighboring institutions, satisfy health network requirements, or comply with reimbursement standards. It is possible that having a particularly high volume of one type of procedure (e.g., spine tumor/spine trauma vs. microdisc/TLIF) could influence a spine center’s likelihood of seeking accreditation, hiring neurosurgeons, and/or being academically affiliated. Still, from our research, we were not able to discern the granularity of the volumes of different types of procedures performed by spine centers. It was presumed that all spine centers offered a full range of commonly performed spinal surgeries (e.g., spinal decompressions) as well as more advanced fusions and deformity corrections (e.g., anterior-posterior cervical fusions, thoracic fusions, and disc arthroplasties).

Although the average distance to an MPC and the number of providers were significantly different between accredited and non-accredited centers, they did not significantly affect the odds of accreditation in the regression model. While neurological surgeon participation, academic center affiliation, and multidisciplinary care provision represent clear and useful distinctions between accredited and non-accredited centers, distance to MPC and average number of providers show such high intra-group variability, diminishing the utility of their comparison; this is to say, there is a large overlap between the two groups. From a patient’s perspective, it would likely appear that accredited and non-accredited centers can be found within similar distances from MPCs and host a similar number of providers, regardless of the results of our analysis.

The question remains as to whether accreditation or self-reporting more reliably identifies centers. While accreditation requirements provide a somewhat objective method for evaluating spine centers based on patient volume and surgical outcomes, among other criteria, solely using these requirements would exclude over 44% of centers that lack accreditation. Given the high degree of similarity in the rates of most reported characteristics, certain aspects of existing spine centers are not covered by current accreditation standards. Characteristics such as the availability of minimally invasive treatments and collaboration with rehabilitation services may contribute to a superior patient experience more directly than other metrics valued by accreditation organizations, like patient volume. Involvement in research could benefit patients by providing a center with more advanced technology and treatment techniques, yet an accredited status does not directly indicate it. Meanwhile, providers seeking to participate in centers may be interested in educating the next generation of physicians and thus be attracted by affiliations with academic medical centers; nonetheless, accreditation does not guarantee this attribute. There may be interest in integrating these characteristics into the parameters for center accreditation, so they are reflected in the designation in the future.

### Limitations

The findings of this study are limited, as they are based solely on publicly available data from Northeastern spine centers. Disparities in accreditation rates and self-reported characteristics could reflect failure by various organizations to publicize their certifications, services, research departments, or other features. Characteristics requiring self-reporting (e.g., minimally invasive procedures, non-surgical treatments, numbers of providers, etc.) were more difficult to verify independently and were likely underrepresented in the observed rates. However, all centers reported certain shared characteristics, and it would stand to reason that they would advertise all pertinent characteristics that would draw prospective patients. Additionally, given this study’s sample’s geographic restriction to the Northeast, regional differences may have led to a center profile that is a significant departure from the average American center. Factors such as the region’s high population density or large number of medical schools could have influenced MPC proximity and rates of affiliation with academic institutions. Finally, because surgical outcomes data are not publicly available via the search methods employed, this component of spine center quality cannot be analyzed within the current study.

## Conclusion

Due to differences between accreditation requirements, the accreditation status of a spine center cannot alone be used to gauge its quality. This heterogeneity poses a potential source of confusion for prospective patients, underscoring the need to standardize accreditation requirements across all spine centers. Ultimately, the Northeast American Spine Center is an organization that provides both non-surgical and surgical treatments for spine conditions, with most centers offering minimally invasive surgical options. Centers typically refer their patients to rehabilitation services, either through in-program physical therapists or closely affiliated outpatient facilities. While most centers are part of an academic medical center, fewer than half participate in research. Only participation by neurological surgeons, provision of multidisciplinary care, and affiliation with an academic medical center are expected at higher rates based on an institution’s formal accreditation status. Additionally, only neurosurgery and academic center affiliation were associated with increased odds of a center having formal accreditation. Providing this initial definition of spine centers will lay the foundation for future research into the standardization of spine centers to improve the quality and expectations of being considered a spine center, as well as the comparison of accredited and non-accredited centers based on volume of patients treated, patient trust, satisfaction with the center, and treatment outcomes.

## Key References

- Burton W, Salsbury SA, Goertz CM. Healthcare provider perspectives on integrating a comprehensive spine care model in an academic health system: a cross-sectional survey. BMC Health Serv Res. 2024;24(1):125. Published 2024 Jan 23. 10.1186/s12913-024-10578-z.
  - ○ This study directly examines the organizational barriers and workforce requirements involved in integrating comprehensive, guideline-concordant spine care within an academic health system.
- Davin S, Lapin B, Udeh B, et al. Health care utilization patterns of patients enrolled in an interdisciplinary program for back pain. N Am Spine Soc J. 2025;21:100584. Published 2025 Jan 14. 10.1016/j.xnsj.2025.100584.
  - ○ This study links an interdisciplinary back-pain program with reduced use of opioids, radiography, and emergency care, providing recent evidence for the value-based rationale behind coordinated spine-care models.
- Wijnen J, Geijselaers MWH, Pont ML, Van't Hullenaar G, Van Oosterwijck J, de Jong J. An Interdisciplinary Multimodal Integrative Healthcare Program for Chronic Spinal Pain and Comorbid Mental Disorders. Psychosom Med. 2024;86(7):603-614. 10.1097/PSY.0000000000001316.
  - ○ This large cohort supports the clinical value of interdisciplinary, multimodal care for patients with chronic spinal pain, reinforcing multidisciplinary care as a meaningful spine-center characteristic.

## Supplementary Information

Below is the link to the electronic supplementary material.

Supplementary Material 1 (DOCX 39.8 KB)

## Data Availability

No datasets were generated or analysed during the current study.
