# Supplementary material for: Spine Centers in Northeastern United States: Assessing Similarities and Disparities to Build more Homogeneity Across the Country
Source: Curr Pain Headache Rep. 2026 Aug 10;30(1):104. doi: 10.1007/s11916-026-01538-9 (PMC13453978; doi:10.1007/s11916-026-01538-9)
Supplement: Supplementary file 1 [file 11916_2026_1538_MOESM1_ESM.docx]

Supplementary Table 1. Accreditation requirements of TJC, DNV, BCBS, and Aetna.

| **Organization** | **Accreditation Name** | **Prior Accreditation By** | **Requirements for Initial Application** | **Annual Reporting Requirements** | **General Requirements** |
| --- | --- | --- | --- | --- | --- |
| TJC**^7^** | Advanced Certification in Spine Surgery (ACSS) | N/A | From past 4 months:   - Surgical site infection rate - Unplanned return visits to OR - Pre-operative and post-operative Patient Reported Outcomes for cervical and lumbar procedures | - Surgical site infection rate - Unplanned return visits to OR - Pre-operative and post-operative Patient Reported Outcomes for cervical and lumbar procedures - Minimum 200 patients served | - Annual site review by TJC representative - Participation in the American Spine Registry (ASR) - Medical equipment training and continuing education for staff - 4 standardized improvement measures - Interdisciplinary collaboration between preoperative, intraoperative, and postoperative teams |
| DNV**^8^** | Advanced Spine Surgery Certification (ASSC) | An organization approved by CMS, certified to act on behalf of CMS, or in association with another DNV-approved entity applying similar requirements | - Quality management metric data - Protocols relevant to the surgical program (enhanced recovery after surgery, selection criteria, discharge planning process, etc.) - Education and competency training for direct patient care staff | 1. Education and competency training for direct patient care staff 2. Most recent healthcare accreditation report 3. Program mission and scope of services 4. Organization chart 5. Number of current patients and pertinent patient information 6. List of patients from the past 6 months 7. 90-day return to surgery rate, 90-day mechanical complication rate, and other performance measures 8. List of names and positions of the program’s interdisciplinary team and management | 1. Patient safety reporting system |
| BCBS**^9^** | Blue Distinction Center (BDC) | For non-ambulatory surgical centers:   1. TJC, DNV, American Association for Hospital and Health Systems (AAHHS), or Center for Improvement in Healthcare Quality (CIHQ)   For ambulatory surgical centers:   1. Prior accreditation by TJC, DNV, AAHHS, American Association for Accreditation of Ambulatory Surgery Facilities (AAAASF), Accreditation Association for Ambulatory Health Care (AAAHC), or Institute for Medical Quality (IMQ) | 1. Meets requirements for prior accreditation 2. Meets general requirements | Meets requirements for prior accreditation  Meets general requirements | Participation of all affiliated providers in the local Blue Plan’s BlueCard PPO  Analytic volume of at least 30 procedures in either:  Primary 1 or 2 level Lumbar Fusion + decompression for Spondylolisthesis  Primary 1 or 2 Level Anterior Cervical Fusion  Reporting of the following for A and B above:  90–day reoperation rate  Unplanned 30-day readmission rate  30–day venous thromboembolism rate  30–day site infection rate  Analytic volume for outcomes  For centers applying for Blue Distinction Center+ (BDC+) certification:  Cost data for lumbar and cervical fusions  Cost data for lumbar and cervical laminectomies  If an ambulatory surgical center or a hospital without an ICU:  Surgical selection criteria developed by a multidisciplinary team  Post-operative spine surgery patient transfer rate  Certification that all inpatient facilities receiving patients from applicant center are designated as Blue Distinction Centers for Spine Surgery |
| Aetna**^10^** | Spinal Institute of Quality (IOQ) | TJC or DNV | Meets requirements for prior accreditation  Meets general requirements | Meets requirements for prior accreditation  Meets general requirements | Registration with ASR  Aetna credentials for all involved inpatient facilities, ambulatory surgical centers, surgeons, and anesthesiologists |
